# Arterial cerebral air embolism occurred during root canal treatment: a case report and literature review

**DOI:** 10.2478/abm-2026-0015

**Published:** 2026-04-30

**Authors:** Beibei Ge, Xinqiang Zhu, Yingxin Ju, Yannan Ma, Yong Song

**Affiliations:** Department of Dentistry, The Affiliated Suqian Hospital of Xuzhou Medical University, Nanjing Drum Tower Hospital Group Suqian Hospital, Suqian, Jiangsu 223800, China 1251533720@qq.com

**Keywords:** air embolism, arterial, cerebral air embolism, endodontic, root canal therapy

## Abstract

**Background:**

Root canal treatment is generally safe, but cerebral arterial air embolism (CAAE) is a rare, life-threatening complication.

**Case presentation:**

A 69-year-old male with apical periodontitis of teeth #31 and #41 suffered sudden loss of consciousness and coma during endodontic treatment. Cerebral computed tomography (CT) revealed multiple intracranial punctate gas densities with cervical and maxillofacial subcutaneous emphysema. Hyperbaric oxygen therapy (HBOT) led to complete neurological recovery.

**Conclusion:**

Clinicians must recognise the risk of arterial air embolism from high-pressure air syringes. Prevention hinges on minimising air pressure and duration, using the lowest effective pressure, and ensuring meticulous haemostasis. Acute neurological deterioration demands immediate imaging and urgent HBOT. Integrating these measures into dental safety standards is essential.

The embolus refers to an obstruction, and the obstruction can be gas. Gases, usually air, accidentally enter the vasculature and form bubbles that block blood flow. It is a serious acute disease that prevents blood flow in arteries and/or veins, leading to ischemia, hypoxia, or even necrosis of tissues or organs. According to the type of blood vessel into which gas enters, it can be divided into venous air embolism and arterial air embolism, and the mechanism and consequences of the two are significantly different. Iatrogenic vascular air embolism is a relatively rare event but may be associated with significant morbidity and mortality. Therefore, early recognition and treatment are essential. The clinical consequences of iatrogenic air embolism depend on the anatomic location and whether the embolism is venous or arterial. Most of the clinically significant ones are caused by arterial occlusion of small vessels, as pulmonary filtration absorbs small air bubbles that enter the venous circulation. However, there is a small proportion of venous air emboli caused by larger bubbles, and patients show more variability [1]. Studies show that an air embolism of 200-300 mL is fatal in humans [2, 3]. Iatrogenic air embolism has been recognized as a medical phenomenon since the late 19th century, yet its clinical significance remains underappreciated [4]. The main factors causing cerebral arterial air embolism (CAAE) are iatrogenic operations [5], especially those involving central veins, thoracic and cardiac surgeries, as well as neurosurgery in a seated position. Trauma and sudden changes in environmental pressure (such as diving and flying) are also important risk scenarios [6]. Early management prioritized positional maneuvers and 100% oxygen inhalation. The therapeutic landscape transformed in the 1970s with hyperbaric oxygen therapy (HBOT) emerging as the definitive intervention through controlled recompression of gas emboli [7]. The subsequent two decades witnessed exponential growth in embolism research, propelled by ultrasound visualization and advanced diagnostic modalities [8, 9]. Pressurized air, used during endodontic procedures, induces severe air embolism by introducing air into the blood vessels through the pterygoid venous plexus located in the gingiva. We report a rare case of fatal air embolism that occurred during routine root canal surgery. Air embolism should be considered a potentially fatal complication of routine surgery. In this report, we review the current literature, discuss common causes, and evaluate management options for air embolism.

## Case presentation

**Figure 1.**
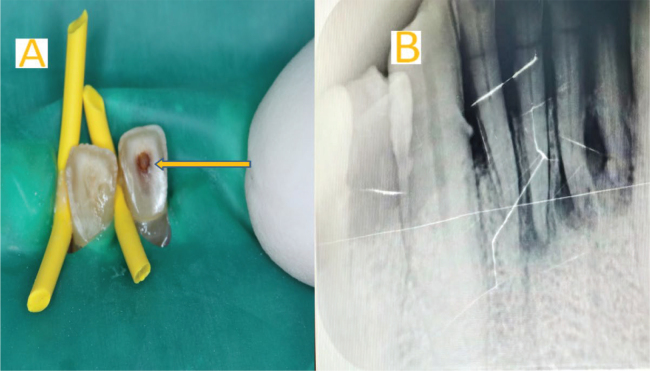
Images prior to root canal treatment. **(A)** Clinical photograph during endodontic treatment of teeth #31 and #41, demonstrating standard operative field setup; **(B)** Preoperative periapical radiograph showing pulp chamber morphology prior to instrumentation.

**Figure 2.**
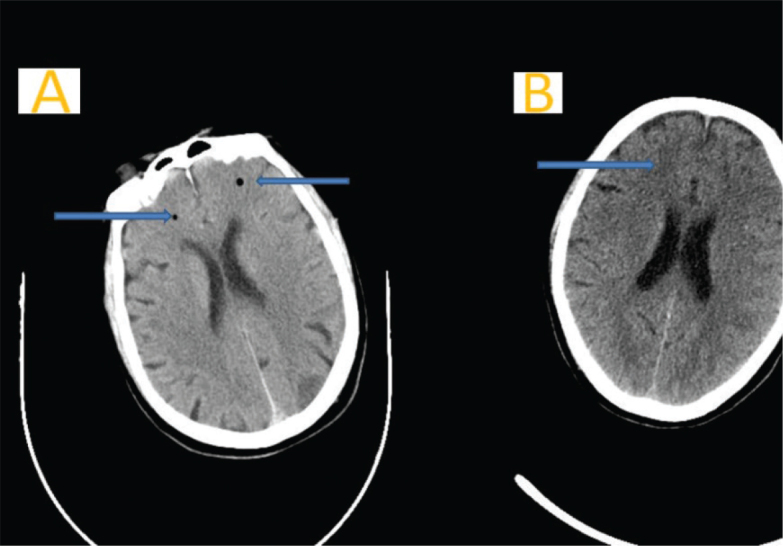
Gas accumulation before and after treatment. **(A)** Axial non-contrast cranial CT scan obtained on September 19 demonstrates intracranial gas accumulation (arrows); **(B)** Follow-up CT 24 h post-HBOT reveals complete resolution of intracranial air collections. CT, computed tomography; HBOT, hyperbaric oxygen therapy.

On September 19, 2024, a 69-year-old man with hypertension on long-term antihypertensive therapy and a 6-year history of coronary artery disease managed with aspirin and rosuvastatin underwent routine root canal treatment for periapical periodontitis of teeth #31 and #41 (**Figure 1**). Preoperative blood pressure was 150/90 mmHg. During the procedure, the patient abruptly developed syncope, respiratory distress, and unresponsiveness, with subsequent vital signs revealing hypertensive crisis (200/160 mmHg) and tachycardia (106 bpm). The patient was urgently transferred to the intensive care unit (ICU) with established intravenous access. Initial evaluation demonstrated sinus tachycardia on electrocardiography and critical hypoxemia (PaO? 75.65 mmHg). Head computed tomography (CT) revealed multiple intracranial gas emboli (**Figure 2A**). Immediate interventions included HBOT, mechanical ventilation, and neuroprotective agents (edaravone combined with citicoline) to address cerebral hypoxia and metabolic crisis. By September 20, follow-up imaging demonstrated significant resolution of pneumocephalus (**Figure 2B**), coinciding with neurological improvement to Glasgow Coma Scale (GCS) 10 with eye-opening to verbal stimuli. Subsequent magnetic resonance imaging on September 21 confirmed complete clearance of intracranial gas. The patient achieved command-following (GCS 14) and resumed oral intake by September 28. A new-onset bilateral sudden sensorineural hearing loss with middle ear effusion emerged on October 2, managed by tympanic membrane puncture and intratympanic dexamethasone administration alongside microcirculation-enhancing therapy. Following 20 sessions of HBOT and coordinated multidisciplinary care, the patient regained baseline neurological function with restored hearing thresholds, ultimately achieving full recovery and discharge.

We conducted the study in accordance with the Declaration of Helsinki, and the patients provided signed informed consent. The research protocol was approved by the Institutional Review Committee and Human Ethics Committee of Suqian Hospital affiliated with Xuzhou Medical University.

## Discussion

CAAE during endodontic procedures involves the use of highspeed dental air turbines (operating at 450,000 RPM) combined with pressurized air-cooling systems. Frictional heat generated during tooth preparation necessitates continuous air-water spray cooling, which may force compressed air into the vascular network through anatomical vulnerabilities in the pterygoid venous plexus. This is closely related to the unique anatomical structure of the dental pulp cavity. Under sustained high-pressure conditions, these introduced gas bubbles can coalesce into clinically significant emboli. Subsequent paradoxical embolization occurs when venous gas emboli traverse intracardiac shunts (particularly the patent foramen ovale) into the arterial circulation, ultimately lodging in cerebral vasculature and triggering ischemic events through mechanical obstruction and endothelial inflammatory cascades [10]. Air can be expelled by diffusion into the alveoli, but easily enters the pulmonary veins if the air volume exceeds 50 mL [10, 11].

Feller-Kopman et al. [12] and Tellides et al. [13] demonstrated a temperature-dependent correlation between heated airflow intensity and gas embolism formation in vascular models, corroborating clinical observations that water-cooled high-speed air turbines may induce vascular air embolism through mechanical insufflation. Additional predisposing factors for CAAE include pre-existing pulmonary comorbidities (particularly chronic obstructive pulmonary disease), semi-recumbent positioning, and hemorrhagic diathesis [11, 14]. In this clinical scenario, the convergence of multiple risk elements proved critical: the patient’s semi-recumbent posture during root canal treatment, combined with sustained elevation of pulpal chamber pressure secondary to prolonged high-speed air turbine operation, created optimal conditions for air entrainment through the periodontium’s vascular network.

Air embolism severity diverges by vascular entry: venous emboli (venous → right heart → pulmonary arteries) may cause subclinical symptoms or, above 50 mL, acute cor pulmonale with hypoxic distress and characteristic “mill-wheel” cardiac sounds. Arterial emboli (systemic → coronary/cerebral) provoke catastrophic end-organ ischemia—myocardial infarction, stroke-like deficits, and multisystem failure via microvascular occlusion. Anatomical embolic routing determines both acute crisis patterns and chronic morbidity profiles.

Positional management differs by embolism type: venous air embolism requires left lateral decubitus with Trendelenburg positioning to trap gas in the right heart, while arterial embolism necessitates right lateral positioning to limit cerebral gas migration [15]. HBOT serves as the definitive treatment, accelerating gas resorption to diminish embolic volume while concurrently enhancing tissue oxygenation and mitigating ischemia-reperfusion injury. Recent advances in microbubble hemodynamics research, through both in vitro models and live vascular studies, have elucidated critical interactions between hemodynamic forces, coagulation pathways, and inflammatory cascades in air embolism pathogenesis. Vascular occlusion by air emboli triggers immediate endothelial activation characterized by pathological, immunological, and prothrombotic inflammatory responses [1]. Gas embolism severity depends on entrained volume, infusion rate, gas composition, and patient positioning during embolization [16].

Cerebral air embolism (CAE), characterized by gas entry into the cerebrovascular circulation, remains a rare yet critical phenomenon in clinical practice [17]. CAE manifests acutely with neurological deficits ranging from focal impairments to coma [18], exemplified by this comatose case. While the Mayo Clinic’s database documents merely 12 arterial CAE cases (predominantly iatrogenic) [18], spontaneous occurrences exist [17]. The condition’s true incidence remains obscured by frequent subclinical presentations and underdiagnosis. HBOT remains the cornerstone therapy for CAE, while adjunctive neuroprotection with lidocaine shows tentative efficacy through incompletely understood mechanisms [19]. While intracranial arterial air embolism is well-documented, our case is notable for its extremely rare cause. Timely and precise intervention resulted in a favorable prognosis. A favorable outcome was achieved through multidisciplinary management of air embolism-related ear nerve damage. Despite the rarity of CAE in endodontic practice, clinicians must maintain high vigilance for this complication when acute neurological symptoms emerge perioperatively, as prompt HBOT intervention significantly mitigates irreversible neurological sequelae and mortality risks. Preventing air embolism is key. Dentists should ensure hemostasis during root canal treatment and, when drying canals, minimize air pressure and limit duration.

## Conclusion

Attention should be paid to the complications of root canal therapy, especially rare complications, in clinical practice. This case also reminds us that during root canal treatment, it is better to dry the root canal, avoid using a high-pressure air gun for a long period, control the lowest effective pressure, and stop bleeding adequately. These protocols are essential to prevent this catastrophic complication.
